# Dipeptide Frequency of Word Frequency and Graph Convolutional Networks for DTA Prediction

**DOI:** 10.3389/fbioe.2020.00267

**Published:** 2020-04-03

**Authors:** Xianfang Wang, Yifeng Liu, Fan Lu, Hongfei Li, Peng Gao, Dongqing Wei

**Affiliations:** ^1^School of Computer Science and Technology, Henan Institute of Technology, Xinxiang, China; ^2^School of Computer and Information Engineering, Henan Normal University, Xinxiang, China; ^3^School of Life Sciences and Biotechnology, Shanghai Jiao Tong University, Shanghai, China

**Keywords:** drug-target binding affinity, dipeptide frequency of word frequency, graph convolutional network, variable importance measures, deep learning

## Abstract

Deep learning is an effective method to capture drug-target binding affinity, but low accuracy is still an obstacle to be overcome. Thus, we propose a novel predictor for drug-target binding affinity based on dipeptide frequency of word frequency encoding and a hybrid graph convolutional network. Word frequency characteristics of natural language are used to improve the frequency characteristics of peptides to express target proteins. For each drug molecules, the five different features of drug atoms and the atomic bond relationships are expressed as graphs. The obtained protein features and graph structure are used as the input of convolution neural network and the input of graph convolution neural network, respectively. A prediction model is established to predict the drug affinity by calculating the hidden relationship. In the KIBA data set test experiment, the consistency coefficient of the model is 0.901, which is 0.01 higher than the existing model, and the MSE (mean square error) of the model is 0.126, which is 5% lower than the existing model. In Davis data set test experiment, the consistency coefficient of the model is 0.895, which is 0.006 higher than the existing model, and the MSE of the model is 0.220, which is 4% lower than the existing model. These results show that our proposed method can not only predict the affinity better than those existing models, but also outperform unitary deep learning approaches.

## Introduction

The discovery processes of the new drug are not only time consuming, but also cost expensively (Roses, 2008). It usually spends about $ 2.6 billion and 10–17 years on research and experimental processes (Yang et al., 2017). One of core method is to find novel targets for existing drugs (Santos et al., 2016) and overcome the current shortage capabilities of drug discovering (Chu et al., 2019). It not only reduces experimental cost, but also greatly shortens drug discovery time (Martin et al., 2018), by eliminating multiple experimental processes such as drug stability (Oprea and Mestres, 2012). How to discover novel target proteins between drugs and targets has become an important task for drug development. And successful identification of drug-target interactions (DTI) is a prerequisite in this task (Ezzat et al., 2019).

High-throughput screening (HTS) experiments are often used to identify the biological activity between drugs and targets, but this method has problems of expensive cost and consumable time (Cohen, 2002). DTI prediction in silicon is one of the effective methods (Liu et al., 2012), and machine learning is a prevalent way (Yan et al., 2019). Support vector machine (SVM) (Keum and Nam, 2017) and random forest (RF) (Wang et al., 2018; Strobl et al., 2019) are often used as predictors in existing research (Olayan et al., 2018). Although these methods are effective, shallow learning models may simplify the relationship between drugs and targeted proteins (Nanni et al., 2020), which are limited by the size of the dataset (Keogh and Mueen, 2009). Deep learning methods have achieved remarkable results in many research areas, such as image processing (Zhou et al., 2020), natural language recognition (Rabovsky and McClelland, 2020), and bioinformatics (Khurana et al., 2018). Its main advantage is that hidden relationships are obtained by calculating of non-linear mapping relationships in original data.

DTI prediction is often considered as a binary classification problem in existing studies (Ban et al., 2019; Yan et al., 2019; Le et al., 2020), that whether or not is a correlation. However, the calculation methods ignore the degree information about DTI, which is the value of binding affinity. Binding affinity provides information about the strength of interactions between drug target (DT) pairs, usually expressed by measures such as dissociation constant (Kd), inhibition constant (Ki), or the half maximal inhibitory concentration (IC50) (Cer et al., 2009). Drug-target binding affinity (DTA) calculated by deep learning algorithms has important research significance.

DeepDTA is a predictive tool for Drug-target binding affinity (Ozturk et al., 2018), which is a Convolutional Neural Network (CNN) that using 1D coding and drug molecular to learn hidden relationships between features and predicting affinity. In order to obtain better model performance, WipeDTA (Öztürk et al., 2019) extracted four text-based information sources to represent proteins and drug structures on the basis of DeepDTA. GraphDTA is an effective prediction model (Nguyen and Venkatesh, 2019), its framework is graph convolutional network that the inputs are graph structure of drugs. OneHot encoding is used to represent protein sequences as input for convolutional neural network. However, these problems what lower expression ability of protein sequence and low prediction ability are caused by the loss of correlation of the OneHot encoding for each residue individually encoded.

In order to overcome the above problems, we propose a novel feature extraction method which is polypeptide frequency of word frequency based on natural language word frequency characteristics to enhance the ability of protein sequence expression. The network model is constructed by merging the graph convolutional network that calculates the graph structure of drugs and the convolutional neural network that calculates the hidden relationship of protein features. The results of output are combined as the input of two hidden layers for regression training and prediction of DTA.

## Data Sets and Feature Extraction

### Data Sets

We use two datasets: KIBA dataset (Tang et al., 2014) and Davis dataset (Davis et al., 2011) (The data sets can obtain from Supplementary Material), as shown in Table 1. KIBA (Tang et al., 2014) was used as a benchmark dataset to evaluate the algorithm model. The Davis dataset (Davis et al., 2011) is lysed selectively using the kinase protein family and associated inhibitors for the dissofarence constant (*K*_*d*_) value, including the affinity of 442 proteins and 68 drugs. We calculate (_*p*_*K*_*d*_) value (as shown in formula 1) regarding the Davis data set use literature processing method to show.

(1) $$\begin{array}{r} pKd=-\mathrm{lg}\left(\frac{Kd}{1e9}\right) \end{array}$$

It can be seen from Table 1 that the number of true interrelationships in the KIBA dataset is about three times that of the statistical interrelationship. KIBA values are calculated based on combinations of different information sources such as IC50, *K*_*i*_, and *K*_*d*_. We used a filtered version of the KIBA data set, where each protein and ligand has ten interactions at least (He et al., 2017).

**Table 1:** Units for magnetic properties number of data sets.

**Data set**	**Number of proteins**	**Number of drugs**	**Number of correlations**
Davis(pKd)	442	68	30,056
KIBA	229	2111	118,254

### Drug Molecular Feature Extraction

**Figure 1:**
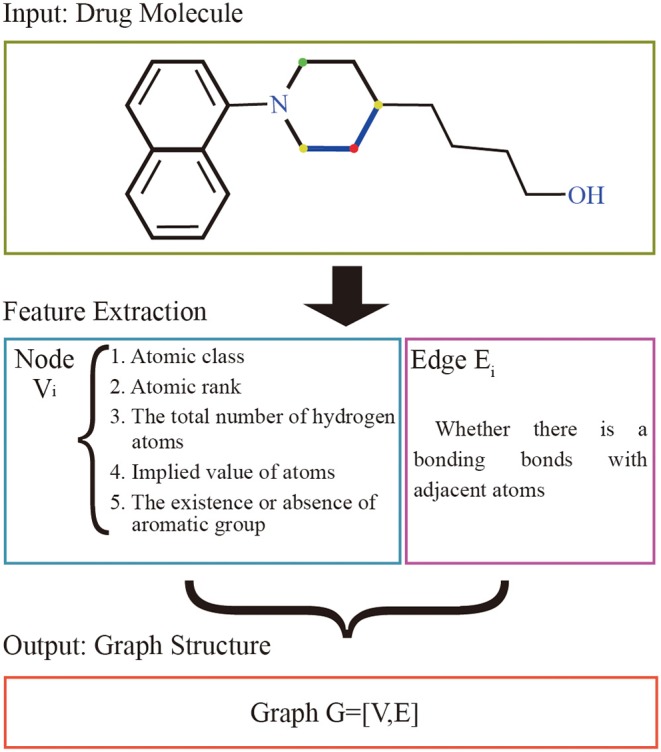
Drug molecular feature extraction process. The input of the extraction process of drugs is the drug molecular structure. Each atom is represented as a node by 5 different characteristics, and the bond between the atom and adjacent atoms is used as the edge set. The red atom has a binding bond with two yellow atoms, and no binding bond with the green atom. The set of nodes and the set of edges are made up of all the atoms together to form the graph structure representing the current drug molecule.

The graphs of the drugs are constructed by using the GraphDTA (Nguyen and Venkatesh, 2019) method. It reflects interactions of internal atom for each SMILES compound. RDkit, open source chemical informatics package (G, 2013), is used to calculate the feature vectors of atom and adjacent atomic connection of drugs. The nodes of the graph represent the features of the drug's atoms, and the bonding bonds between the atoms are represented by the edges. The features vectors of the drug atomic are made up of five characteristics: atomic class, atomic rank, the total number of hydrogen atoms, implied value of atoms, and the existence or absence of aromatic groups. The atomic rank is the sum of the number of the bond between the current atom and neighboring atoms and the number of hydrogen atoms. The edge of graph represents the connection relation of adjacent atoms. The overall process is shown in Figure 1.

### Protein Sequence Feature Extraction

#### Protein Sequence Representation

The first order-structure vectorization is a prerequisite for data analysis of protein sequences, formula 2 is used to discretize the primary structure of the protein.

(2) $$\begin{array}{r} S_{n}=R_{1}R_{2}R_{3}.\ldots R_{i}\ldots R_{L},\;\left(n\leq K\right) \end{array}$$

where *S*_*n*_ is the nth protein data, *R*_*i*_ is the *ith* amino acid residue in the protein sequence, *K* is the number of protein sequences in the data set.

#### Polypeptide Frequency of Word Frequency

Term frequency-inverse document frequency (TF-IDF) algorithm plays an important role in Natural Language Processing (NLP) (Kaur and Jatinderkumar, 2019). TF-IDF is consisted of Term Frequency (TF) and Inverse Document Frequency (IDF). The algorithm of polypeptide frequency *F* (as shown in Formula 3) is similar to the calculation process of TF in bioinformatics.

(3) $$\begin{array}{r} F={\left(v_{1},v_{2},v_{3},\ldots ,v_{25^{n}}\right)}^{T} \end{array}$$

Where, *n* is the number of 25 residues contained in the polypeptide, thus 25^*n*^ different polymers are formed by dehydration condensation, *v*_*i*_ represents the frequency of the *ith* feature of the polypeptide. The formula for *v*_*i*_ is as follows.

(4) $$\begin{array}{r} v_{i}\text{=}n_{u}/\overset{25^{n}}{\underset{u=1}{\sum }}n_{u}=n_{u}/\left(L-1\right) \end{array}$$

where *L* represents the length of the protein sequence, *n*_*u*_ *r*epresents the occurrence times of uth dipeptide signature in the protein sequence.

IDF is the reversion document frequency to increase important weight of TF, as specified in formula 5.

(5) $$\begin{array}{r} IDF=\mathrm{lg}\left(\frac{N}{w_{i}}\right),\left(i=1,2,3,4,\ldots ,25^{n}\right) \end{array}$$

where, in bioinformatics, *N* is the number of protein sequences in the data set, and *w*_*i*_ is the number of protein sequences which contain the *ith* polypeptide. From the formula, it can be known that the occurrence frequency of current words is inversely proportional to IDF, so TF-IDF algorithm will assign a lower feature for the high-frequency words. Which is not suitable for bioinformatics calculation. Therefore, we propose the polypeptide frequency of method word frequency, which can avoid this problem by only calculates the word frequency. As shown in formula 6:

(6) $$\begin{array}{r} WF={\left(wf_{1},wf_{2},wf_{3},\ldots ,wf_{i},\ldots ,wf_{25^{n}}\right)}^{T} \end{array}$$

where, *n* is the number of residues that make up the polypeptide, and *wf*_*i*_ is the frequency of the *ith* polypeptide of word frequency, as shown in formula 7.

(7) $$\begin{array}{r} wf_{i}=\frac{w_{i}}{N}\times \frac{p_{i}}{L-1} \end{array}$$

where, *w*_*i*_ is the number of protein sequences containing the *ith* peptide, *N* is the total number of proteins contained in the data set, *p*_*i*_ is the number of times that the *ith* peptide appears in the current protein, and *L* is the number of residues contained in the current protein.

### Network Model Construction

**Figure 2:**
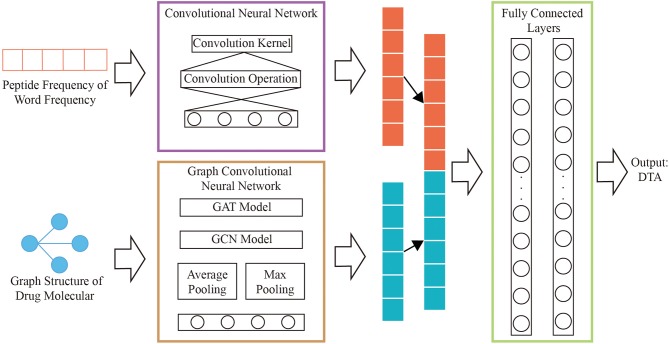
Network structure diagram.

A novel model that combining graph convolutional neural networks and convolutional neural networks are designed to regressively predict DTA. The multi-layers graph convolutional neural network is used to obtain the hidden relationships of drug graphs. The hidden relationships of the polypeptide frequency of word frequency are obtained through the convolutional neural network calculation. The output results of the two networks are combined as the input of fully connected layers. The complete process is shown in Figure 2.

### Graph Convolutional Neural Network of Drug

We use the improved four types of graph convolutional neural networks by GraphDTA to discover potential relationships for the graph structure of drug features, which are GCN (Kipf and Welling, 2017), GAT (Veličković et al., 2018), GIN (Xu et al., 2019), GAT-GCN (Nguyen and Venkatesh, 2019). The linear connected layer that the inputs are results of graph convolutional neural networks maps to a 128-dimensional features vectors, which is consistent with the size of feature vectors for protein.

The GCN model is originally proposed by Kipf and Welling (2017) as a graph structure learner for semi-supervised classification. In order to meet the requirements of regression in our work, three graph convolutional units are made that include a GCN layer and a ReLU activation layer. The number of output channels is 78, 156, 312, respectively. And a fully connected layer of 1,024 neurons is created, the results are mapped to a 128-dimensional features vector in the output layer.

Graph Isomorphism Network (GIN) is an improved algorithm based on GCN. Injective aggregation updates the parameters and performs the feature vector mapping to obtain better model performance. The network model of the five-layer GIN layer is designed, and each GIN layer consists of two linear calculations with an output size of 32. The input and output layers are mapped into 128-dimensional features vectors.

Graph Attention Network (GAT) is different from the GCN model, the difference is that it calculates the corresponding hidden information for each node and introduces an attention mechanism when computing its neighboring nodes. The network model is designed using two GAT layers. In the first layer, the number of output channel is 78, and the number of attention nodes is 10. In the second layer, the number of output channel is 128, and the number of attention nodes is 1. The results are input to the output layer, which map to a 128-dimensional features vector.

Based on the GAT and GCN models, GAT- CCN integrates the advantages of the two models in series to obtain better model performance. The output channel of the GAT layer is 78, the number of attention nodes is 10. And the output channel of the GCN layer is 780. And a fully connected layer of 1,500 neurons is created, results are mapped to a 128-dimensional features vector in the output layer.

### Convolutional Neural Network of Protein

Convolutional neural network is used to obtain hidden relationships in vector of protein features. A 1D convolutional neural network is designed by analyzing the characteristic structure of protein word frequency and polypeptide frequency. The model contains a convolution kernel that the size is 32. The result of the convolution calculation is input to the fully connected layer for mapping to 256 neurons, keeping the size of the drug, and protein consistent.

We concatenate the feature vectors of proteins from convolutional neural networks and the feature vectors of drugs from graph convolutional neural networks. And they are input to two fully connected layers with 512 and 128 neutrons, respectively. And set the batch size to 512 and the learning rate to 0.00005.

## Results and Discussion

### Performance Evaluation

In this work, the datasets are divided into two parts: training set and test set. That is, 80% of data instances are used for training, and 20% are for testing the models. The performances of our model are comprehensively compared by several experiment using evaluation metrics such as Concordance Index (CI), Mean Squared Error (MSE), as well as Pearson correlation coefficient. The evaluation indicators are consistent with WideDTA and GraphDTA. the performance of the predicted models of output continuous values is evaluated by CI, the formula is as follows.

(8) $$\begin{array}{r} CI=\frac{1}{Z}\underset{\delta x>\delta y}{\sum }h\left(bx-by\right) \end{array}$$

where *b*_*x*_ is the prediction value for the larger affinity δ_*x*_, *b*_*y*_ is the prediction value for the smaller affinity δ_*y*_. Z is the normalization constant, *h(m)* is the step function, and as shown in the following formula:

(9) $$\begin{array}{l} h\left(m\right)=\{\begin{array}{c} 1,\;if\;m>0\\ 0.5\;if\;m=0\\ 0\;if\;m<0 \end{array} \end{array}$$

MSE is often used for the difference between the predicted value and the actual value vector, and it's an important index for evaluating regression models, the formula is as follows.

(10) $$\begin{array}{r} MSE=\frac{1}{n}\overset{n}{\underset{k=1}{\sum }}{\left(b_{k}-\delta_{k}\right)}^{2} \end{array}$$

where *n* is the number of data in the data set of KIBA or Davis, and other parameters have the same meaning as above.

Pearson correlation coefficient evaluates the difference of the affinity between the true value and the predicted value, the formula is as follows.

(11) $$\begin{array}{r} pearson=\frac{\mathrm{cov}\left(p,y\right)}{\sigma \left(p\right)\sigma \left(p\right)} \end{array}$$

where *cov* indicates the co-variance, *p* is predicted values, *y* is original values, σ represents the standard deviation.

### Contrast Experiments and Analysis of Different Characteristics

In this study, we introduced polypeptide frequency of word frequency that was a novel way of protein feature extraction. The peptide frequency includes several methods. For every protein sequence, we calculated the word frequency characteristics and frequency of single peptide, dipeptides, as well as tripeptides. And different graph convolutional network models were designed to predict drug-target binding affinity. The results of comparative experiment are shown in Table 2.

**Table 2:** Comparative experimental results of word frequency feature of many different peptides.

**Graph neural network model**	**Peptides**	**KIBA**			**Davis**		
		**MSE**	**CI**	**Pearson**	**MSE**	**CI**	**Pearson**
GAT	1	0.758	0.372	0.323	0.740	0.649	0.436
	2	0.176	0.868	0.873	0.231	0.899	0.698
	3	0.187	0.858	0.823	0.244	0.861	0.659
GIN	1	0.427	0.696	0.569	0.472	0.802	0.634
	2	0.148	0.881	0.856	0.222	0.894	0.687
	3	0.151	0.871	0.851	0.239	0.882	0.685
GCN	1	0.803	0.431	0.341	0.834	0.408	0.337
	2	0.127	0.898	0.864	0.223	0.894	0.697
	3	0.151	0.873	0.846	0.247	0.887	0.691
GAT_GCN	1	0.624	0.798	0.698	0.743	0.644	0.434
	2	**0.126**	**0.901**	**0.893**	**0.220**	**0.899**	**0.701**
	3	0.191	0.852	0.839	0.224	0.896	0.693

*The bold values are maximum*.

When the protein sequence is represented by the word frequency dipeptide frequency and the GAT_GCN model, the model is the best predictor for 3 evaluation metrics yielding a CI of 0.901, a MSE of 0.126, and a Pearson of 0.893 in KIBA data set, and yielding a CI of 0.895, a MSE of 0.220 and a Pearson of 0.701 in Davis data set. When word frequency dipeptide frequency was used to represent protein sequences, compared with the second best GCN model, the CI and Pearson of GAT_GCN model in KIBA data set are increased by 0.03 and 0.029, respectively, and the MSE value decreases by 0.01. Compared with GAT and GIN models, the CI values of GAT_GCN model are 0.033 and 0.020 higher, the MSE values are reduced by 0.050 and 0.022, and Pearson values are increased by 0.020 and 0.037, respectively. In the Davis data set, the CI value of GAT_GCN model is same with GAT model as the next-highest model, the MSE value is reduced by 0.011, and Pearson is increased by 0.003. The CI value of the GAT_GCN model is 0.005 higher than the GCN and 0.002 higher than GIN. The MSE values are decreased by 0.003 and 0.005, and the Pearson values are increased by 0.004 and 0.006, respectively. So, the GAT_GCN model has the best performance in these four models.

When the GAT_GCN model is used as a graph calculator, compared with the word frequency single peptide frequency and the word frequency tripeptide frequency, the CI values of word frequency dipeptide frequency in the KIBA dataset are higher by 0.103 and 0.049, the MSE values are reduced by 0.498 and 0.065, respectively. In the Davis data, CI values are 0.251 and 0.003 higher, the MSE values are decreased by 0.578 and 0.004, and Pearson values are increased by 0.195 and 0.054, respectively. The word frequency dipeptide frequency characteristics can also obtain the optimal index when combined with GIT, GAT, GCN models in the KIBA and Davis data sets, indicating that the word frequency dipeptide frequency characteristics have the best performance index compared to other characteristics.

### Word Frequency Comparison Experiment

**Figure 3:**
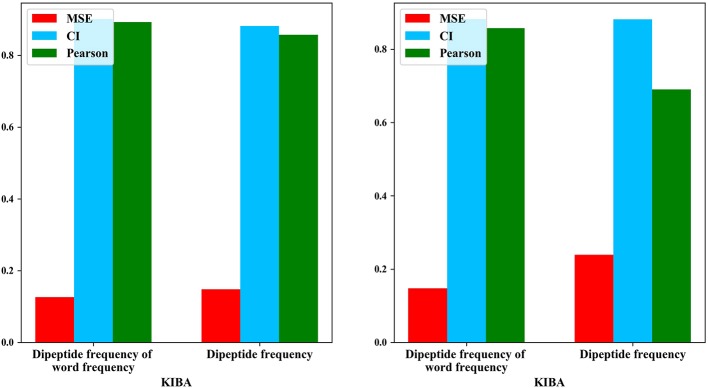
Comparison results of dipeptide features.

We also compared the differences in dipeptide frequencies with or without word frequency characteristics. The results are shown in Table 3 and Figure 3.

**Table 3:** Comparison results of dipeptide features.

**Features**	**KIBA**			**Davis**		
	**MSE**	**CI**	**Pearson**	**MSE**	**CI**	**Pearson**
Dipeptide frequency of word frequency	**0.126**	**0.901**	**0.893**	**0.220**	**0.899**	**0.701**
Dipeptide frequency	0.148	0.882	0.857	0.239	0.881	0.690

*The bold values are maximum*.

After adding the word frequency characteristics based on the dipeptide frequency, the MSE decreased by 0.022 and the CI and Pearson increased by 0.009 and 0.033 in the KIBA data set, and MSE decreased by 0.019 and the CI and Pearson increased by 0.018 and 0.009 in Davis data set. This shows that the dipeptide frequency of word frequency is more conducive to the prediction of the classifier than the dipeptide frequency, and has better represented ability for protein sequences.

### Analysis of Protein Features

**Figure 4:**
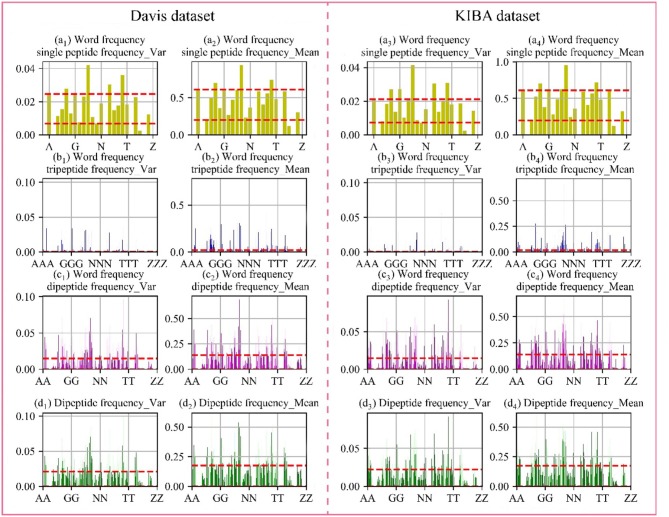
Frequency chart of word frequency peptides. The X-axis is the peptide amino acid combination, and the Y-axis is the word frequency polypeptide frequency score. **(a)** Are the scores of the single peptide frequency of word frequency, **(b)** are the scores of the tripeptide frequency of word frequency, **(c)** are the scores of the dipeptide frequency of word frequency, and **(d)** are the scores of the dipeptide frequency. The red lines represent the upper and lower quartiles. The first and second columns are the Davis data set, and the first and second columns are the KIBA data set.

Through the analysis of comparative experiments, we found that the model was obtained the best performance metrics when dipeptide frequency of word frequency be used to represent protein sequences. For every protein, we calculated the mean and variance in the Davis and KIBA datasets, respectively. The results are shown in Figure 4.

In the Davis dataset and the KIBA dataset, the distribution of score are basically same. The single peptide frequency of word frequency features scores are mainly concentrated between 0.20 and 0.61, and the variances are mainly concentrated between 0.007 and 0.220. Although there is a high features scores and large variance, the features have too high differences in the vectors of feature. And the number of features is only 25 dimensions, which contributes less to the spatially specific division of the model. Although the tripeptide frequency of word frequency features have a huge number of 15,625 dimensions, the scores are mainly distributed below 0.018, and the variances are mainly distributed below 0.003. The features have small differences between data, and there are a lot of features with value of 0. The scores of dipeptide frequency of word frequency characteristic mainly have a distribution range between 0 and 0.14, and the variances have a main distribution range between 0 and 0.0149, which has a good score and data difference.

**Figure 5:**
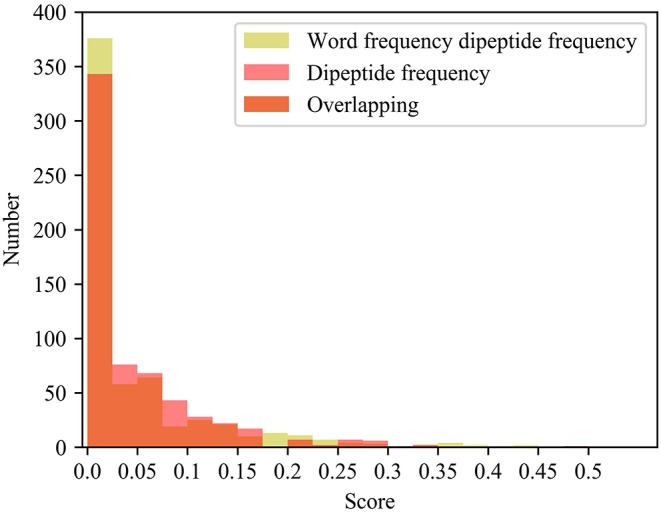
Histogram of frequency distribution. Yellow represents the histogram of the frequency distribution of the word frequency dipeptide, red represents the histogram of the frequency distribution of the word frequency dipeptide, and orange represents the overlap between the two.

Compared with the dipeptide frequency of word frequency, the score of dipeptide frequency are mainly distributed below 0.17, and the variances are mainly distributed between 0 and 0.021. Although it has a good score, the difference is high in vectors of feature, as same as the word frequency single peptide frequency. In order to discover the difference between the frequency characteristics of dipeptide and word frequency dipeptide, we draw a histogram of the frequency distribution of the two and perform overlapping processing, the results are shown in Figure 5.

After adding the word frequency characteristics, the number of dipeptide frequency of word frequency features is less than that of the dipeptide frequency in the score intervals [0.025, 0.175] and [0.250, 0.300]. And the number of dipeptide frequency of word frequency features is more than that of dipeptide frequency features in the score intervals [0, 0.025] and [0.175, 0.250]. Dipeptide frequency features distribution is at [0, 0.35], and the dipeptide frequency of word frequency features distribution is at [0, 0.45], and the interval range is greater and more continuous. It shows that the frequency characteristics of words can play a role in reducing non-significant features and improving score difference.

### Analysis of Variable Importance Measure

**Figure 6:**
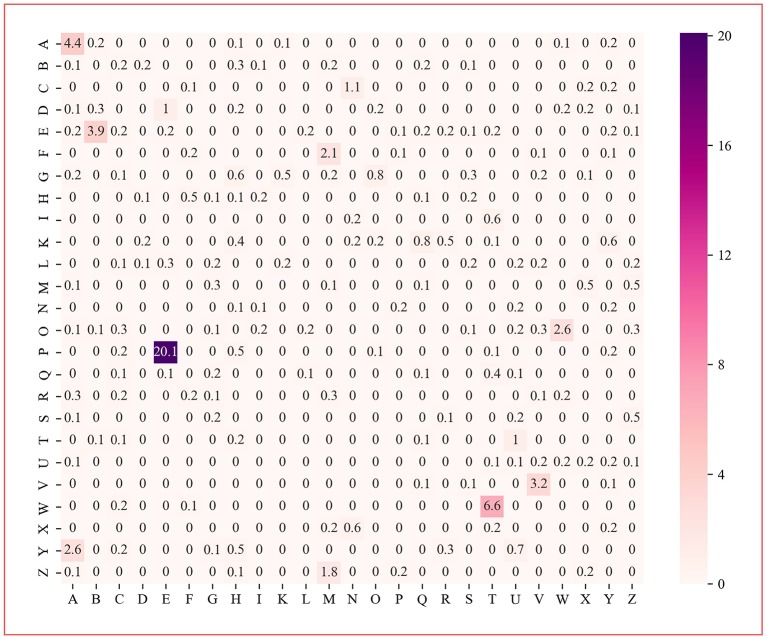
Dipeptide frequency of word frequency VIM score. Its X-axis and Y-axis are 25 kinds of amino acids. Each point represents the importance score of the corresponding dipeptide frequency of word frequency characteristic variable. The color from white to purple represents the score from low to high.

**Figure 7:**
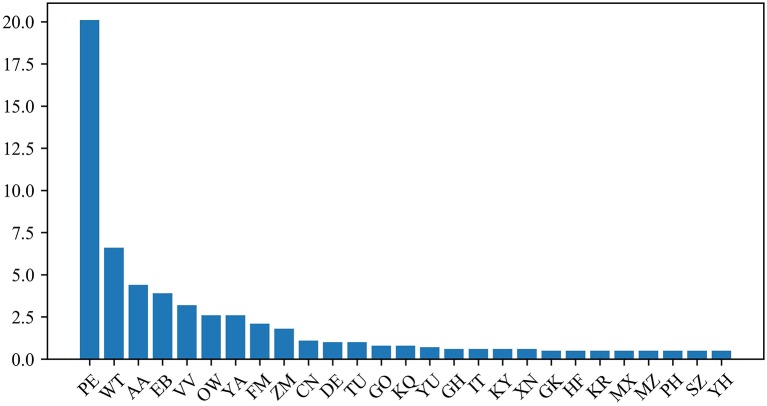
Features ranking diagram with contribution >0.5%.

The protein dipeptide frequency of word frequency is composed of 625-dimensional features. The Variable Importance Measures (VIM) is used to analyze the contribution of each feature. In bioinformatics, Random Forest (RF) is a commonly used classification and regression model (Belgiu et al., 2016). And its unique advantage is to calculate VIM (Rawi et al., 2018), compared with other machine learning algorithms such as support vector machine (SVM). We used the RF model containing 10,000 decision trees to obtain the VIM score of features in the dipeptide frequency of word frequency, as shown in Figure 6. Features of non-zero VIM score have 199 dimensions, indicating that there's much noise in the vectors of features. The 27-dimensional features what a contribution >0.5% are listed in Figure 7. The top five dipeptide frequency of word frequency features are PE (20.1%), WT (6.6%), AA (4.4%), EB (3.9%), and VV (3.2%). This shows that PE (the combination of proline and glutamic acid) is significantly related to the affinity prediction, which is about three times of the second highest WT (the combination of tryptophan and threonine) and much larger than other combinations.

### Comparison of Existing Models

The predictor of our work was compared with state-of-the-art methods what DeepDTA, WideDTA, and GraphDTA by using an independent test set in Davis and KIBA. The results are shown in Table 4.

**Table 4:** Algorithm comparison experiment results.

**Features**	**KIBA**		**Davis**	
	**MSE**	**CI**	**MSE**	**CI**
DeepDTA	0.194	0.863	0.261	0.878
WipeDTA	0.179	0.875	0.262	0.886
GraphDTA	0.139	0.891	0.229	0.893
This model	**0.126**	**0.901**	**0.220**	**0.899**

*The bold values are maximum*.

Our method outperformed state-of-the-art methods with two main quality metrics as CI and MSE in Davis and KIBA. Compared with the DeepDTA and WipeDTA models, our model reduced the MSE by 0.068 and 0.041, which increased the CI by 0.048 and 0.026, respectively in the KIBA dataset. And MSE decreased by 0.061 and 0.062, CI increased by 0.021 and 0.013, respectively, in the Davis data set. It shows that the graph neural network model with input as the graph structure of the drug can obtained better performance. Our method outperformed the GraphDTA model using the same graph convolutional neural network, the MSE decreased by 5% (0.007) and the CI increased by 0.01 in the KIBA data set, the MSE decreased by 4% (0.009) and the CI increased by 0.006 in the Davis data set. It shown that the dipeptide frequency of word frequency has better ability to express targeted proteins and can obtain better prediction models than 1D coding.

## Conclusion

The DTA plays an important role in the discovery of new drugs. Dipeptide frequency of word frequency which is a novel feature extraction method is employed to represent protein sequences by natural language processing techniques. In addition, we use graphs to represent the drugs structure where the nodes is constructed by five different features and the edges represent atomic bond relationship. A network model is constructed, it is consisted of three parts: convolution neural network, graph convolution neural network, and fully connected layers. Convolutional neural network that input is dipeptide frequency of word frequency is to calculate hidden relationships of protein data. Graph Convolutional neural network is constructed to calculate hidden relationships for the graphs of drugs. The results of the two network models are mapped and combined to the fully connected layer predicting DTA. The results of peptide frequency comparison experiment showed that the dipeptide for the division of the spatial relationship was better than the monopeptide and tripeptide, so that the model performance can be obtained better. The results of the dipeptide frequency comparison experiment showed that adding word frequency characteristics for the dipeptide frequency can reduce the features difference. In comparison experiment of state-of-the-art model, our model has improved performance comparing with DeepDTA and WideDTA models, which indicating that the graphs can express the structure of drugs better. And experimental results show that our model has better performance than the GraphDTA model using graph convolutional neural network. In the KIBA dataset, MSE decreased by 5% (0.007) and CI increased by 0.01, and in the Davis dataset, MSE decreased by 4% (0.009) and CI increased by 0.006. It showed that the frequency characteristics of word frequency dipeptide could represent protein sequences better. Through the analysis of protein features, we observed that the vector have certain differences and intensity when the average score of the features is below 0.014 and the variance score is below 0.015, which are more conducive to the spatial division. In the analysis of variables importance, it was found that PE, WT, AA, EB, and VV had a high contribution to model prediction, among which PE (the combination of proline and glutamate) was highest by 20.1%. Besides drug discovery, the Dipeptide frequency of word frequency proposed in this work may also be applied in other field to represent protein sequence. Thus, it has the practical significance.

## Data Availability Statement

The datasets [Dives] for this study can be found in the [Comprehensive analysis of kinase inhibitor selectivity] [https://www.nature.com/articles/nbt.1990]. The datasets [KIBA] for this study can be found in the [Making Sense of Large-Scale Kinase Inhibitor Bioactivity Data Sets: A Comparative and Integrative Analysis] [https://pubs.acs.org/doi/10.1021/ci400709d].

## Author Contributions

XW and YL designed the study and wrote the manuscript. FL translate manuscript. HL and PG analyzed data and drawn illustrations. DW provides theoretical guidance on Drug-Targets. All authors have read and approved the final manuscript.

### Conflict of Interest

The authors declare that the research was conducted in the absence of any commercial or financial relationships that could be construed as a potential conflict of interest.
